# Accuracy of commercial electronic nicotine delivery systems (ENDS) temperature control technology

**DOI:** 10.1371/journal.pone.0206937

**Published:** 2018-11-05

**Authors:** Seyed Ahmad Reza Dibaji, Suvajyoti Guha, Aarthi Arab, Bruce T. Murray, Matthew R. Myers

**Affiliations:** 1 Center for Devices and Radiological Health, U.S. Food and Drug Administration, Silver Spring, Maryland, United States of America; 2 Center for Tobacco Products, U.S. Food and Drug Administration, Silver Spring, Maryland, United States of America; 3 Department of Mechanical Engineering, Binghamton University (State University of New York), Binghamton, New York, United States of America; University of New South Wales, AUSTRALIA

## Abstract

**Objectives:**

For electronic nicotine delivery systems (ENDS), also commonly called e-cigarettes, coil temperature is a factor in the potential production of toxic chemical constituents. However, data are lacking regarding the temperatures that are achieved in the latest generation of these devices. Fourth-generation ENDS are capable of producing heating coil temperatures well above e-liquid boiling points, and allow the user to monitor and set the heating coil temperature during a puff. In this study, we evaluate the accuracy and consistency of the temperature measurement and control settings for different brands of fourth-generation ENDS.

**Methods:**

A study was performed using three commercially available, fourth-generation ENDS. The atomizer coil temperatures were obtained from the device (using the EScribe software) reading and from thermocouples attached to the coils during simulated puffing conditions. In addition, aerosol temperatures were measured inside the atomizer and at the mouthpiece.

**Results:**

Measured temperatures varied widely across samples taken from the same brand. For example, thermocouple measurements for one unit were 40 Celsius (°C) below the 300 °C set point, while another unit of the same brand exceeded the set point by more than 100 °C. We observed a significant variation in temperature (approximately 100 °C) along the length of the coil in some cases.

**Conclusions:**

The possibility of wide temperature variation across ENDS samples, as well as variations between maximum coil temperatures and internal temperature readings, may have implications for studies that seek to determine correlations between coil temperature and toxin generation.

## 1. Introduction

The use of ENDS (electronic nicotine delivery systems) has grown rapidly in the last ten years [1]. Although the basic components (battery, heater coil, liquid reservoir, wick material, etc.) are common across device generations, their complexity has changed substantially. The fourth-generation ENDS have batteries capable of supplying up to 300 Watts (W) of electrical power as well as advanced electronic circuitry that monitors and maintains the coil temperature during puffing. These devices differ vastly from earlier generations of ENDS, which supply power in the 1–15 W range and are not capable of temperature control.

Based on studies of earlier generations of ENDS, several factors can affect the performance of these devices and the creation of chemical byproducts. These factors include: a) design characteristics (e.g., type of heating coil, wick material, air flow control), b) e-liquid constituents, c) power level and other operating conditions, and d) user behaviors, such as puffing topography [2, 3]. Recent studies indicate a clear trend toward higher vaporization temperatures capable of breaking down chemical constituents in e-liquids (including the carrier liquids propylene glycol (PG) and vegetable glycerin (VG)) and yielding substantial amounts of aldehydes and acrolein [4–6], which can lead to cancer or other serious health effects [7–9]. For example, a recent study suggested that even a small power increase from 4.3 W to 9.6 W, with a concomitant increase in temperature, can result in very large increases of formaldehyde, acetaldehyde and acetone levels [10]. Another investigation found that, compared to 70 °C coil temperatures, a 325 °C temperature can result in a 3-fold increase in formaldehyde concentration [6]. Yet another study, found that a temperature increase from 270 °C to 320 °C can cause an 8-fold increase in formaldehyde formation and a significant amount of acetaldehyde formation [4].

Because levels of harmful or potentially harmful constituents (HPHCs) such as formaldehyde, acrolein, and acetaldehyde [2] have been found at varying ENDS device temperature levels and at varying quantities, it is important to fully understand the relationship between temperature and HPHC levels. It is also important to know the temperature at critical locations (including coil hot spot) within the ENDS and how these temperatures relate to the display temperature determined by the device measurement system. Once these factors are understood, this information can be used to better understand the effects of ENDS on public health. Previous studies have measured coil temperature using thermocouples [11] or infrared (IR) thermography [10]. In one study using thermocouples, the thermocouple junctions were not attached to the coil; therefore, the precise location of the temperature measurement within the atomizer was difficult to ascertain [11]. The use of thermocouples to measure the coil or vapor temperatures poses additional difficulties, such as the location of the thermocouples with respect to the coil. Improper positioning can interfere with device operation. Also, performing IR thermography is difficult to do while the ENDS device is in use with puff flow, since the coils cannot be exposed to the camera. In a recent study [12], the coil temperature of a second-generation e-cigarette device was measured, for different powers (2.4 W to 13.3 W), coil wetness, and e-liquid compositions. Thermocouple measurements were compared with values determined via IR thermography. Measurements were conducted without puff flow and with the mouthpiece removed. The impact of airflow was not considered. The IR camera results demonstrated nonuniform temperature across the heating coil, though the spatial temperature distribution across the coil was not provided. The maximum temperatures measured by thermocouples were reported to be 51% to 96% of the coil maximum temperature recorded by IR camera. The difference was attributed to possible variation in the location of thermocouples attached to the coils.

The main purpose of this study is to measure the temperature at various locations within fourth-generation ENDS products that are fully operational, and relate these temperatures to the display temperature determined by the device measurement system. An additional goal is to validate a method using thermocouples and cement adhesives for measuring atomizer coil temperature, for use in future thermal studies of high-power ENDS. We performed a comprehensive set of experiments on ENDS products, pairing the atomizers with “box mods” (base unit comprising the batteries, electronic circuitry and controls) to allow for temperature and power control.

## 2. Materials and methods

### 2.1. ENDS design features

**Fig 1:**
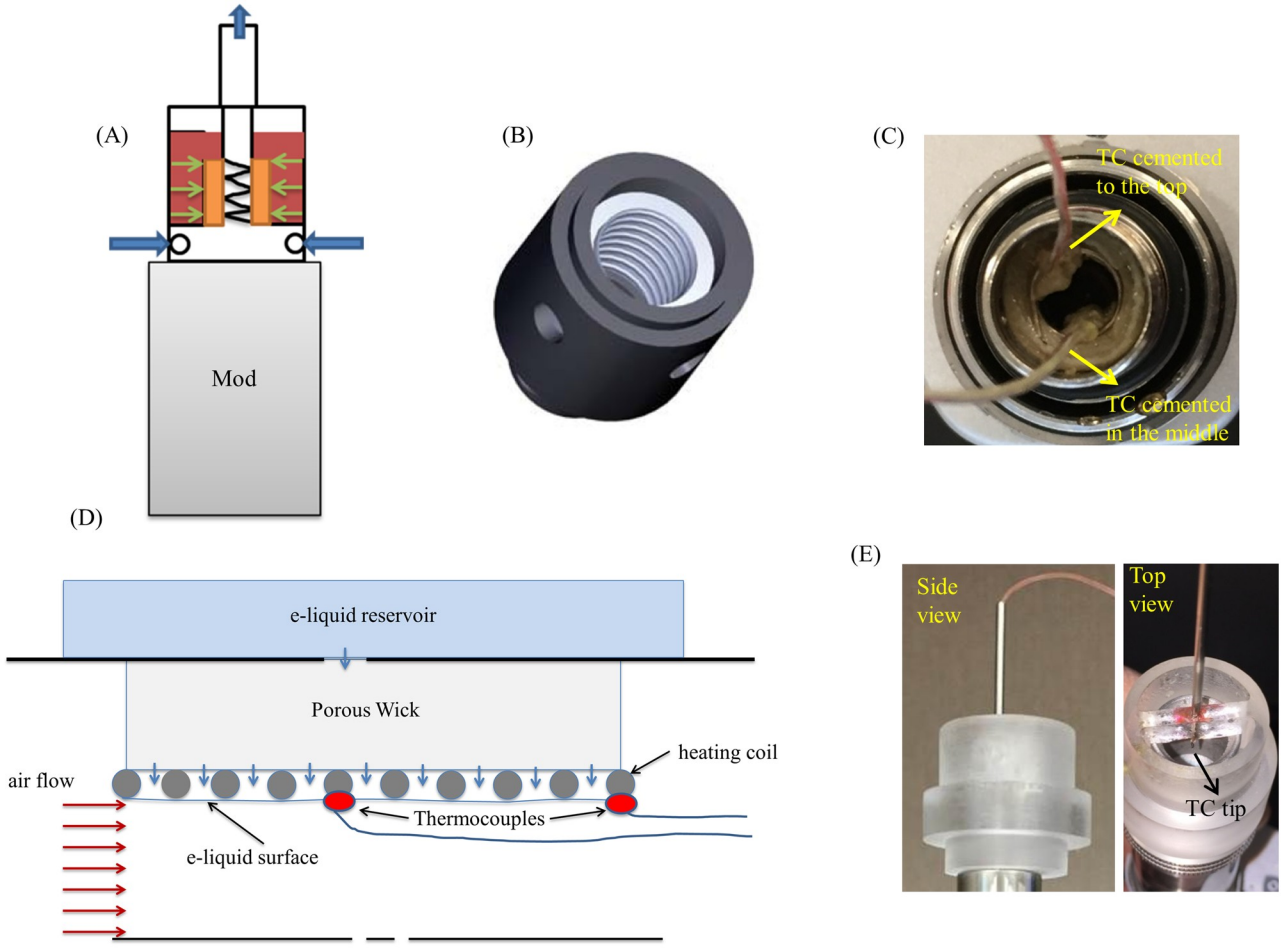
ENDS schematic and temperature measurement set up. (A) schematic of recent generation e-cigarette (blue arrows indicate air flow direction); (B) 3D drawing of internal atomizer with visible coiled wire heating element and central air flow channel. (C) Two thermocouples (TC) cemented to the top and middle sections of heating coil for measuring temperature; (D) Axisymmetric schematic view of typical atomizer configuration showing approximate locations of attached thermocouples. (E) A constructed fixture for measuring aerosol smoke temperature. The fixture holds the thermocouple in place in the center of the flow path.

A schematic drawing of a typical fourth-generation ENDS is shown in Fig 1A. The vaporizer unit attaches to a box mod via a standard threaded coupling, which provides the electrical connection. The vaporizer unit consists of its housing, a glass or plastic tank containing an e-liquid, an atomizer, and a mouthpiece. Together, the atomizer, tank unit, and box mod constitute the ENDS. The removable atomizer unit consists of a wire coil surrounded by a porous wick (cotton, silica, or metal fibers), which is inserted into a metal annular outer shell. Holes in the outer shell serve as inlets for the e-liquid that flows from the tank. The housing provides a central cylindrical air flow channel through the atomizer and the mouthpiece (Fig 1A and 1B).

#### 2.1.1. DNA200 and EScribe temperature measurement

The ENDS control circuitry measures the voltage, resistance and current as a function of time during operation. The temperature is determined by the resistance variation with temperature. All ENDS brands tested in this study utilize the DNA 200 microchip (Evolv, LLC) a popular microprocessor that can measure and control temperature. The DNA 200 operation can also be controlled externally using Evolv’s EScribe software. The mods can be operated in Temperature control mode (TCM) that allows the user to specify the maximum coil temperature during operation. The available temperature control range is 100–300 °C. The user can also set the maximum power (wattage level) attained in TCM operation. Enabling the TCM feature on fourth-generation ENDS, which are capable of supplying up to 300 W, may reduce the risk of inhaling harmful chemicals by preventing the heating coil from reaching extremely high temperatures. In a recent study [12], the maximum heating-coil temperature of a second-generation e-cigarette device was reported as high as 1008 °C for a dry coil (no e-liquid), using a power of 13.3 W. The devices also have a power control mode which is not addressed in the current study. The temperature control and monitoring features are based on the coil material’s variation of resistance with temperature. For the range of 20–400 °C, a linear relationship is normally assumed [13]:

$$R\;=\;R_{0}[1+\alpha (T-T_{0})],$$ (1)

where R is the resistance at temperature T, R_0_ is the reference resistance at temperature T_0_, and α is the temperature coefficient of resistance. Only a limited number of wire materials, including nickel (Ni), stainless steel and titanium, have a sufficiently high positive thermal coefficient (PTC) of resistance for temperature control. A graph comparing the PTC of nickel, stainless steel, and titanium is provided in the supporting information (S1 Fig). Ni and Ni alloys have the largest variation of resistance with temperature and are thus most widely used. In this study, we tested three ENDS brands with the same Ni 200 alloy and cold ohm resistance of 0.15 Ω.

### 2.2. Temperature measurement techniques

#### 2.2.1. Coil temperature

We used 36-gauge K-type thermocouples (5SC-TT-K-36-36, Omega Engineering, Inc.) to measure the coil and aerosol temperatures. These thermocouples can measure up to 1250 °C, have an accuracy of 2.2 °C, and have a response time of less than 0.1 s. To ensure good contact and reduce electrical interference, the thermocouples were secured to the coil with thermally conductive cement (OB-400, Omega Engineering, Inc.). Because of the cement’s thermal conductivity and thermal mass, the response time of thermocouples increased by about 1 s, but the accuracy of the measured steady-state temperature was not affected. In order to validate the use of cement to attach thermocouples to the heating coil without any electrical interference, we conducted a set of experiments using a thermocouple cemented to the surface of a ceramic hot plate (11-100-49SH, Fisher Scientific). The temperature of the hot plate was varied from 50 °C to 500 °C in increments of 50 °C. At each increment, once the hot plate temperature reached steady-state, we compared the hot plate surface temperature recorded by the cemented thermocouple, the hot plate set temperature, and the temperature measured by an infrared thermometer (62 Mini, Fluke Corporation). The IR thermometer operating range is -30 °C to 500 °C with an accuracy of ± 1.5 °C or ± 1.5% of reading (whichever is greater). The cemented thermocouple and IR thermometer measurements are in close agreement (S2 Fig). At lower temperatures (≤ 200 °C), the measurements agree within 3%, while at higher temperatures (≥ 200 °C) the agreement is within 6%.

The volume of cement surrounding the thermocouple junction was minimized to prevent obstruction of the air flow (Fig 1C). For atomizers with small air passageways, we used only one thermocouple; for those with wider air passageways, we attached two thermocouples, one positioned at the top end of the coil (atomizer exit) and another at the mid-section of the coil (schematics shown in Fig 1D). A data acquisition system collected the thermocouple readings as a function of time (OMB-DAQ-3000, Omega Engineering, Inc.).

#### 2.2.2. EScribe temperature measurements

As noted above, the mods in this study use the DNA 200 chip. The mod measures and records voltage, cold ohm (initial value of the coil resistance), live ohm (resistance), and calculates current, power and temperature during operation. The mod is connected to a laptop computer via its micro USB port. The EScribe software controls the device and records data in real-time. Temperature data was sampled every 0.2 s. Using the software, we also recorded the cold resistance and monitored the live resistance throughout each puff and compared that with the values calculated by Eq (1). There was a close agreement (≤ 3% on average) between the recorded resistances by the software and Eq (1).

#### 2.2.3. Aerosol temperature

In addition to the coil temperature, we measured aerosol (emitted vapor) temperature using a thermocouple located at the centerline of the flow path at different axial locations. We constructed a fixture, attached to the mouthpiece (Fig 1E), to hold the thermocouple in place at the center of the air flow path, while also allowing the thermocouple to move axially between the atomizer and the exit of the mouthpiece.

### 2.3. Experimental study

The experimental protocol had 3 components: 1) perform direct measurements of the atomizer heating coil temperatures via attached thermocouples; 2) simultaneously obtain and compare measures of the coil temperature from thermocouples and the EScribe software and 3) measure the aerosol temperatures within the atomizer and at the mouthpiece.

We conducted experiments using three commercially available tank atomizers, referred to here as Atomizer A, B, and C. We used the same DNA 200 mod unit for all the tests, because initial testing showed no significant variation in the temperature control function between mods (S3 Fig and S1 Table). We performed tests using three different units of each atomizer brand to assess variability within the brand. Most coil temperature measurements were made with no air flow through the device, or at a flow rate of 55 mL/s. The 0 mL/s and 55 mL/s rates represent limiting values [3, 14], i.e. flow rates occurring during actual use of ENDS are likely to reside between these extreme values. In a study by the E-Cigarette Task Force [15], a square 55 mL puff of 3 s in duration was recommended for adoption as an interim standard vaping regime for e-cigarette testing. In other studies on puffing behavior [16–17], the average puffing duration was shown to vary from 2.5 s to 3.9 s. As a point of reference, a 3.5 s puff duration with a 55 mL puff volume corresponds to an average flow rate of ~15.7 mL/s. To more completely assess the variability with flow rate, we also used a flow rate of 25 mL/s in some experiments. For the tests with air flow, an air stream metered by a mass flow controller (ALICAT MC-20SLPM) was delivered through connecting tubes to the tank atomizer inlets (Fig 1A). The air flow was supplied continuously at the adjusted flow rate during the puff. We used a square-shaped puff profile over a duration of 10 s. We activated the devices for 10 s to capture the full range of puff durations, including those occurring in vaping competitions. The first 3–5 s of the 10 s puff are considered to be representative of actual usage.

For all the experiments, we used an e-liquid composition of 65% VG, 35% PG, and 3 milligram/milliliters (mg/mL) of nicotine. We chose this mixture because of its popularity within the vaping community. Vegetable glycerin produces a denser vapor at a sufficiently high coil temperature. Propylene glycol provides greater flavor intensity and a soothing feeling to the throat, according to some members of the vaping community [18–20]. The boiling point of the VG/PG mixture was calculated to be approximately ~221 °C, using a boiling point composition diagram [21]. The 300 °C temperature used in most of the experiments assured that the e-liquid boiled during the procedures.

## 3. Results

### 3.1. Temperature and power variation over the duration of a puff

**Fig 2:**
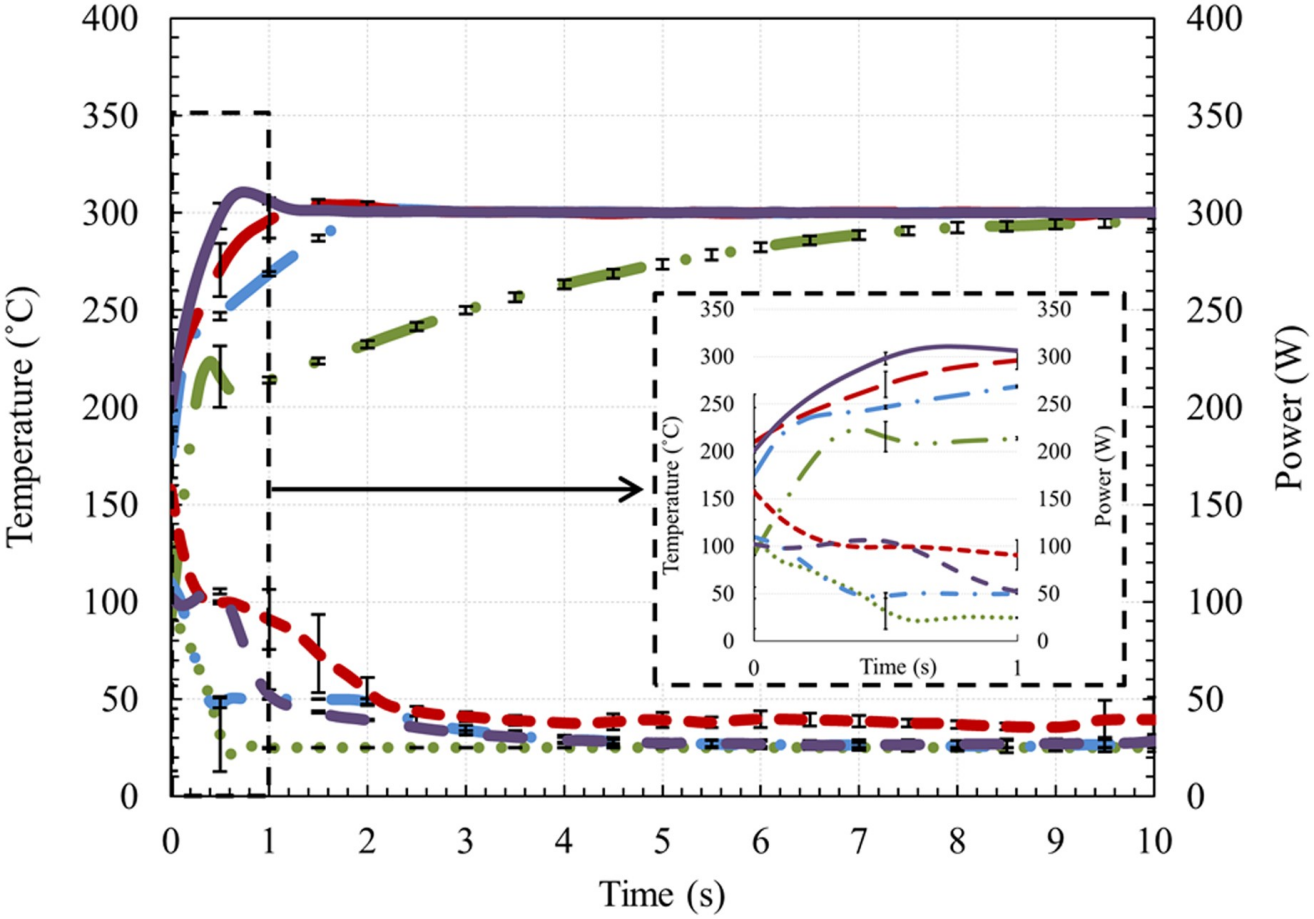
Coil temperature and corresponding power recorded by the EScribe software for the 25 W, 50 W, 100 W, and 200 W power settings. Long dash dot dot green line: temperature for 25 W setting, long dash dot blue line: temperature for 50 W, long dash red line: temperature for 100 W, solid purple line: temperature for 200 W, round dot green line: power for the 25 W setting, dash dot blue line: power for the 50 W setting, square dot red line: power for 100 W setting, dashed purple line: power for the 200 W setting. The inset image shows a magnified section of the graph from 0–1 s; the inset uses the same line descriptions in the caption above.

Fig 2 shows the mod temperature and power output for Atomizer A under TCM operation, with the temperature set at 300 °C. Four power limits, 25, 50, 100 and 200 W, were used. A pre-heat setting, in which a short spike of power (typically 120–180 W) is applied before each puff to preheat the coil, was used in each of the experiments. We did not supply any air flow to the unit during this first set of experiments. The data shown in Fig 2 for each power setting is the average of three sets of measurements from a single test sample (Atomizer A) and the error bars correspond to the standard deviation of three trials. At 100 and 200 W levels, the coil temperature first reached the 300 °C set point in less than 1 s, overshot the 300 °C (set point temperature) value, and then settled back down to 300 °C after 1 to 2 s. For the lower power settings (25 and 50 W), the device took considerably more time for the coil to reach the 300 °C set point. A typical puff is 3 to 4 s; therefore, the temperature set point was not always reached during a puff duration for the lower power levels. In addition to the temperature traces, Fig 2 shows the power variation by the device to keep the coil temperature at 300 °C during the 10 s puff, for each of the pre-set powers of 25 W, 50 W, 100 W, and 200 W. The puff started with an application of the pre-set value for the power. As the coil temperature reached the 300 °C set point, the control circuitry reduced the power to keep the temperature at the set point. After 3 s, the power was reduced to ~40 W for the pre-set power of 200 W, while it was reduced to ~25 W for the pre-set powers of 25 W, 50 W, and 100 W.

### 3.2. Atomizer coil axial temperature variation

**Fig 3:**
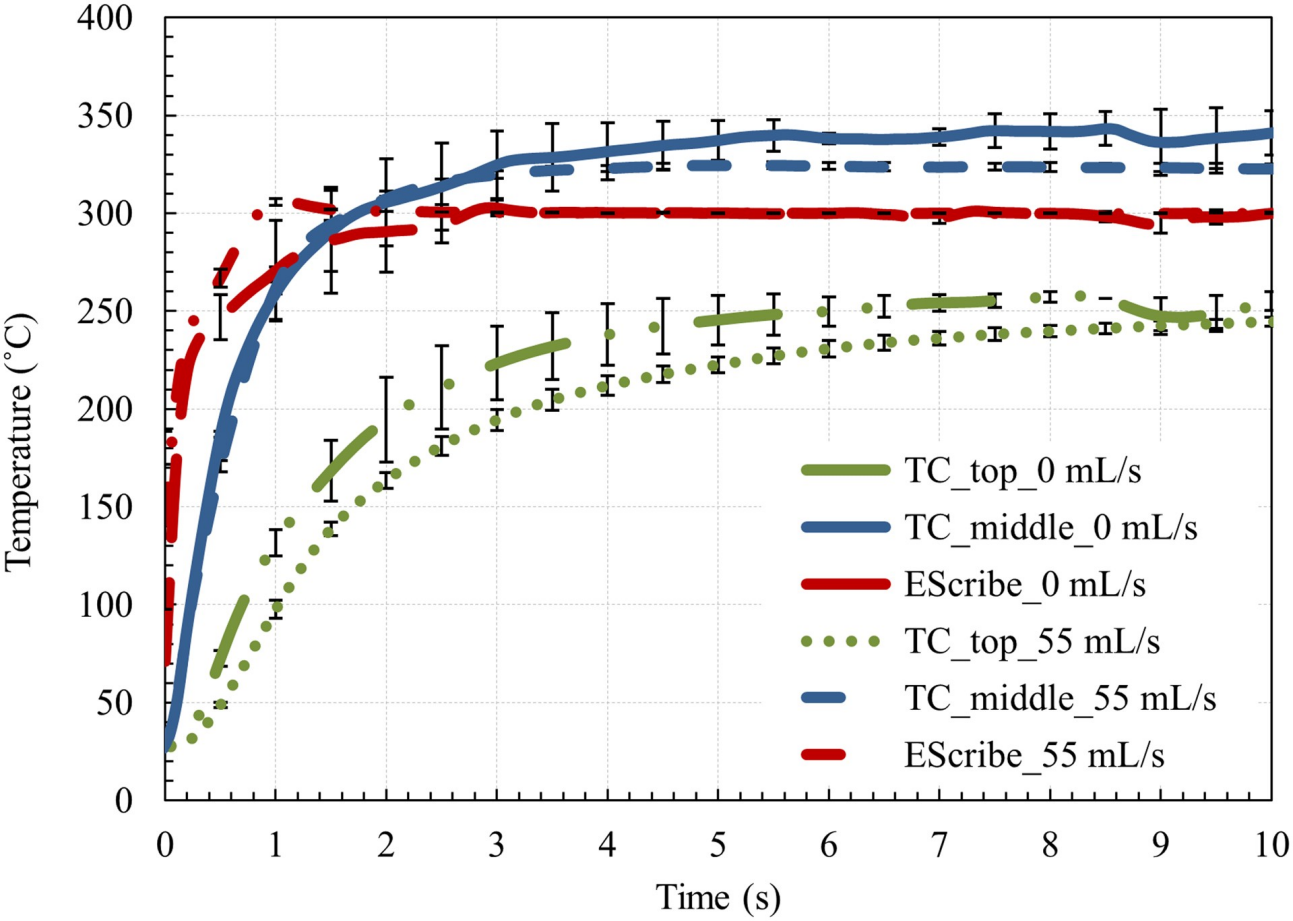
Coil temperature recorded by TCs and EScribe for two flow rates of 0 mL/s and 55 mL/s. Long dash dot dot green line: temperature measured at the top section of the coil for the air flow rate of 0 mL/s, solid blue line: temperature measured in the middle section of the coil for the air flow rate of 0 mL/s, long dash red line: temperature measured by EScribe for the air flow rate of 0 mL/s, dot green line: temperature measured at the top section of the coil for the air flow rate of 55 mL/s, dash blue line: temperature measured in the middle section of the coil for the air flow rate of 55 mL/s, dash dot red line: temperature measured by EScribe for the air flow rate of 55 mL/s.

Fig 3 displays the temperature measured using thermocouples at the middle and top of Atomizer A. We used TCM with a set point of 300 °C and the power limit set point of 100 W. Coil temperatures were measured with no air flow and with an air flow of 55 mL/s. The error bars in Fig 3 correspond to the standard deviation of three measurements from a single test sample (Atomizer A). The temporal resolution of measurements was 0.1 s. However, the error bars are shown at every 0.5 s for all the temperature plots to provide better clarity. The temperature at the middle and top of the coil differed by more than 100 °C during the first few seconds. Additionally, the middle of the coil exceeded the set point of 300 °C for both air flow rates, while the temperatures at the top of the coil were significantly below the set point. By comparison, the EScribe temperature, which is an average over the entire length of the heating coil, was right on set point after an initial adjustment period. The adjustment involved a slight overshoot for the 55 mL/s air flow.

### 3.3. Variation between brands and within a brand

We obtained thermocouple measurements on a minimum of three sample units from each atomizer brand (A, B, and, C). Because of the small diameter of atomizer C, we could only attach one thermocouple at the top end of the coil. For this atomizer, we tested six sample units. Results are summarized in Table 1. The end-of-puff temperature was below the 300 °C pre-set temperature in 26 of the 33 cases and above the pre-set temperature in 7 cases. The average end-of-puff temperature was 278 °C, with a standard deviation of 50 °C. For the same pre-set temperature of 300 °C, the minimum temperature observed was 188 °C, and the maximum was 417 °C.

**Table 1:** Recorded maximum temperature by thermocouple (TC) for different brand of atomizers (Ni 200, 0.15 Ω) with: Temperature control = 300 °C, power = 100 W, puff duration = 10 s, airflow rates of 0 mL/s and 55 mL/s.

Atomizer brand	Atomizer sample	Puff duration (s)	Average (over 3 trials) peak temperature (°C)			
			Flow rate: 0 mL/s		Flow rate: 55 mL/s	
			Top	Middle	Top	Middle
**A**	A1	10	258 ± 2	***343 ± 9***	245 ± 2	***324 ± 2***
	A2	10	282 ± 4	276 ± 1	275 ± 3	236 ± 6
	A3	10	254 ± 10	276 ± 6	211 ± 1	249 ± 3
**B**	B1	10	262 ± 2	285 ± 2	213 ± 5	269 ± 5
	B2	10	232 ± 3	280 ± 1	188 ± 6	262 ± 2
	B3	10	244 ± 7	286 ± 3	231 ± 5	278 ± 6
**C**	C1	10	269 ± 17	✘	262 ± 5	✘
	C2	10	***417 ± 40***	✘	***398 ± 9***	✘
	C3	10	***352 ± 12***	✘	***324 ± 10***	✘
	C4	10	275 ± 5	✘	✘	✘
	C5	10	***339 ± 44***	✘	✘	✘
	C6	10	292 ± 11	✘	✘	✘

^✘^ indicates that there was no measurement.

***Bold Italics*** indicate that the measured temperature exceeded the pre-set temperature value.

### 3.4. Aerosol temperature measurement

**Fig 4:**
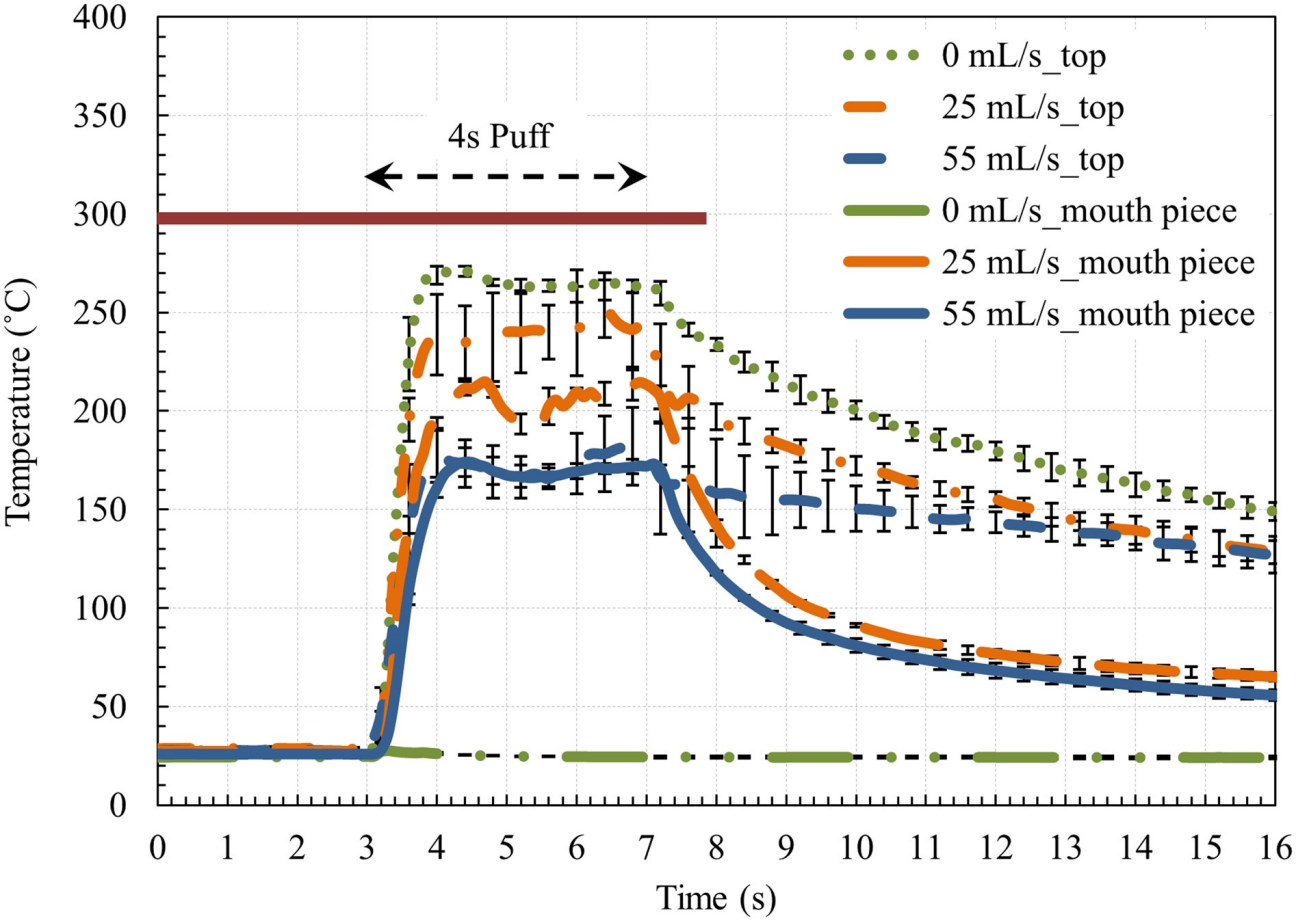
Aerosol temperature at the top section of atomizer and at the mouth piece of the e-cigarette. Dot green line: temperature at the top section of the atomizer for the air flow rate of 0 mL/s, dash dot orange line: temperature at the top section of the atomizer for the air flow rate of 25 mL/s, dash blue line: temperature at the top section of the atomizer for the air flow rate of 55 mL/s, long dash dot dot green line: temperature at the center of the mouth piece for the air flow rate of 0 mL/s, long dash orange line: temperature at the center of the mouthpiece for the air flow rate of 25 mL/s, solid blue line: temperature at the center of the mouthpiece for the air flow rate of 55 mL/s.

Similar to the data provided in Fig 3, each temperature value shown in Fig 4 is the average of three measurements for a single test sample, and the error bars were shown at every 0.5 s to provide better clarity, though the temporal resolution of measurements was 0.1 s. As shown in Fig 4, at all flow rates, the aerosol temperature (vaping product temperature which measured at the mouthpiece outlet) sharply increases at the start of the puff, corresponding to the surge in power sent to the coil (Fig 1). Since there was no cement applied in the aerosol temperature measurement, there was no delay in the thermocouple response time as there was with the coil measurements. At 0 mL/s (no air flow), no aerosol/vapor exited the mouthpiece and, therefore, no heat was convected through the device. As a result, the temperature measured by the thermocouple was almost the same as the background (ambient) temperature. The aerosol temperature remained below the set point (300 °C) and decreased significantly at the end of the puff. The mouthpiece was substantially cooler than the top of the atomizer, except at the high flow rate, where there was essentially no temperature difference between the two locations. The average and maximum aerosol temperatures decreased with increasing flow rate.

## 4. Discussion

This work addresses three important prerequisites in order to correlate temperatures generated by fourth-generation ENDS with the toxins they produce. With more than 200 W of power available to ENDS users, the temperature that the coil can reach is near that of pyrolysis in traditional cigarettes. The first prerequisite to understanding the implications of these high temperatures is the development of a methodology for accurately measuring the temperature distribution within the devices. The second is the quantification of the accuracy and repeatability of the temperature measurements made by the ENDS device itself. The third is understanding how coil temperatures correspond to aerosol temperature.

Because of the high-power capability of the fourth-generation ENDS, very high coil temperatures were obtained very quickly. In some cases, the coil temperature exceeded the boiling point of the e-liquid in less than a second (as seen in Fig 2). The rapid influx of heat into the e-liquid at the high-power settings makes investigation into the possibility of e-liquid breakdown important. At the lower power levels, also featured in Fig 2, the rise to the set point temperature was gradual, despite a large amount of applied power at the beginning of the puff. Even in temperature control mode, if the wattage level is not high enough and the puff is too short, the coil will not have time to reach the maximum temperature setting.

Coil temperature measurements (as seen in Fig 3) indicated that the temperature at the center of the coil was considerably higher (on the order of 100 °C for the test conditions) than the temperature at the end of the coil. This temperature differential between the middle and the end of the coil is to be expected, given that the electrical energy is dissipated uniformly throughout the wire, but the ends of the coil dissipate thermal energy to the more massive, lower-temperature housing. In addition to the difference between mid- and end-coil temperatures, there is a difference between the mid-coil temperature and the set point temperature. The temperature at the center of the coil can considerably exceed (as much as 40 °C for the conditions depicted in Fig 3) the set point temperature displayed by the EScribe software. Future studies that choose to obtain the temperature from EScribe determined values should be aware that these temperatures represent an average of the overall coil temperature. This is important because the actual maximum coil temperature can affect the expected constituent yields in ENDS aerosols and it is important to know the actual temperature distribution of the coil and how much the actual maximum temperature varies from average temperature provided by the device.

The variation in the internal resistance-based temperature measurements (obtained via EScribe) between atomizers of the same brand (Table 1) was considerable. For Atomizer A, the standard deviation (averaged over the two flow rates and two thermocouple locations) of the temperature measurements for the three samples was 34 °C. For Atomizer B, the standard deviation for the three samples was 12 °C, and for Atomizer C, it was 63 °C. These large variations prevented any conclusion from being drawn regarding the difference between brands in actual temperature produced for the same set point. An important finding in the present study is that actual temperatures may be affected by an unforeseen factor—the manufacturing consistency and batch of the atomizer—which implies the need for standard development in coil manufacturing.

For the non-zero puff flow rates shown in Fig 4, the aerosol temperature at the top of the atomizer was found to be significantly below the pre-set temperature, owing to the mixing of the vapor heated by the coil with the cool air flowing through the atomizer. Still, the aerosol temperatures reported here were considerably higher than those reported for conventional cigarettes [22] and second-generation e-cigarettes [23]. In the study by Sleiman et al. [23], the emitted vapor mean temperature inside the mouthpiece was reported as ~39 C° for an EGO vaporizer (2.6 Ω coil resistance) at 4.8 V, which corresponds to the power of ~8.9 W. The heating coil temperature was not reported in this study. In our study on the fourth-generation of ENDS, the aerosol temperature measurements were conducted while the power and temperature control were set to 100 W (almost 10 times the applied power by Sleiman et al. [23]) and 300 °C, respectively. As noted by Sleiman et al. [23], the temperature of the coil and amount of heat transferred to the vapor depends on different factors, including the power output, puff duration, airflow velocities, coil geometry, the amount of liquid carried by the wick, and the heat capacity of the e-liquid. For the same setting, the coil and aerosol temperature can vary widely from device to device. The long-term effects to the extra-thoracic region from such high temperature exposure is unknown. It should be noted that the aerosol temperatures could increase from those shown in Fig 4 depending on the axial location of the measurement. The heating coil is located at the outer border of the cylindrical chamber (Fig 1); therefore, due to proximity to the heat source (heating coil), gas particles will experience higher temperatures closer to the heating coil and away from the center.

We assumed that the temperature pattern for consecutive puffs would be the same as the ones shown in Fig 3 (for coil temperature) and Fig 4 (for smoke temperature). However, the heating coil and the aerosol maximum temperatures can vary between the consecutive puffs depending on the puff duration and inter-puff interval. Increasing the puff duration as well as reducing the inter-puff interval result in the higher maximum heating coil and aerosol temperatures for the subsequent puffs.

The clinical and regulatory implications of the results of this investigation await an analyses of the toxins generated by ENDS devices, under the wide range of performance exhibited by the devices under the conditions of this study.

## 5. Conclusions

The user-controlled temperature control feature of the fourth-generation of ENDS can result in the coil temperature exceeding the e-liquid boiling point very rapidly, within a fraction of the puff duration. Also, the resistance-based temperature measurements of the devices themselves correspond to an average value for the temperature along the coil. The temperature can vary significantly along the coil due to the nature of the heat transfer and vaporization of e-liquid. The temperature measured by the device can substantially underestimate the temperature in the center of the coil.

A wide variation in measured coil temperatures between atomizer samples was observed, with some samples of a given brand manifesting thermocouple temperatures 40 °C below the 300 °C set point, and other samples of the same brand generating more than 100 °C in excess of the set point. This shows that not only are there concerns with the accuracy of the device’s own temperature reading but also that variability within a brand should be accounted for in risk assessments. At the 300 °C set point, the measured aerosol temperature by the thermocouple at the mouthpiece was found to be as high as 160 °C. Future work will involve physical and chemical characterization as well as computational modeling at various temperatures and e-liquid compositions to thoroughly characterize the spatially complex temperature field, and its effect on the aerosol size distribution and potential toxic product yields.

## Supporting information

S1 Fig Electrical Resistivity as a function of temperature for Stainless Steel, Titanium, and Nickel. Note: the presented data were collected from the manufacturer website (www.steam-engine.org).
(TIF)

S2 Fig Hot plate temperature measured by cemented TC and IR thermometer. (TIF)

S3 Fig (A) Coil temperature recorded by TC at the top-section of the atomizer B for three different mods, (B) Coil temperature recorded by TC in the mid-section of the atomizer B for three different mods. Solid red line: recorded temperature for mod_1, dash blue line: recorded temperature for mod_2, and dot green line: recorded temperature for mod_3. Note: All the three different tested mods (referred to here as mod_1, mod_2, and mod_3) had the same DNA 200 microchip. The power and temperature control were set at 100 W, and 300 °C, respectively. The temperature measurements were made with no airflow for the puff duration of 10 s.
(TIF)

S1 Table Corresponding statistic (Anova) for TC measurements presented in S3 Fig at the top and mid-section of atomizer B for the three different applied mods (mod_1, mod_2, mod_3) at the selected time points of: 1 s, 3 s, 5 s, 7s, and 9 s. Note: An analysis of variance for 5 different time points showed that in 8 of the 10 comparisons, no significant difference was discernable. Essentially, the variation within tests of a single mod exceeded the variation between mods.
(DOCX)

## References

[1] Kaisar MA, Prasad S, Liles T, Cucullo L. A decade of e-cigarettes: Limited research & unresolved safety concerns. Toxicology. 2016; 365: 67–75. 10.1016/j.tox.2016.07.020 27477296 PMC4993660

[2] Gillman IG, Kistler KA, Stewart EW, Paolantonio AR. Effect of variable power levels on the yield of total aerosol mass and formation of aldehydes in e-cigarette aerosols. Regul Toxicol Pharmacol. 2016; 75: 58–65. 10.1016/j.yrtph.2015.12.019 26743740

[3] Talih S, Balhas Z, Eissenberg T, Salman R, Karaoghlanian N, El Hellani A, et al Effects of User Puff Topography, Device Voltage, and Liquid Nicotine Concentration on Electronic Cigarette Nicotine Yield: Measurements and Model Predictions. Nicotine Tob Res. 2015; 17(2):150–17. 10.1093/ntr/ntu174 25187061 PMC4837998

[4] Wang P, Chen W, Liao J, Matsuo T, Ito K, Fowles J, el al. A Device-Independent Evaluation of Carbonyl Emissions from Heated Electronic Cigarette Solvents. PloS One. 2017; 12(1): e0169811 10.1371/journal.pone.0169811 28076380 PMC5226727

[5] Jensen RP, Luo W, Pankow JF, Strongin RM, Peyton DH. Hidden formaldehyde in e-cigarette aerosols. N Engl J Med. 2015; 372(4): 392–394. 10.1056/NEJMc1413069 25607446

[6] Talih S, Balhas Z, Salman R, Karaoghlanian N, Shihadeh A. "Direct Dripping": A High-Temperature, High-Formaldehyde Emission Electronic Cigarette Use Method. *Nicotine Tob Res*. 2016; 18(4): 453–459. 10.1093/ntr/ntv080 25863521 PMC6220833

[7] IARC working group on the evaluation of carcinogenic risks to humans. Formaldehyde, 2-Butoxyethanol and 1-tert-Butoxypropan-2-ol. IARC Monogr Eval Carcinog Risks Hum. 2006; 88:1–478. 17366697 PMC4781641

[8] Wang HT, Hu Y, Tong D, Huang J, Gu L, Wu XR, et al Effect of carcinogenic acrolein on DNA repair and mutagenic susceptibility. *J Biol Chem*. 2012; 287(15): 12379–12386. 10.1074/jbc.M111.329623 22275365 PMC3320987

[9] Feng Z, Hu W, Hu Y, Tang MS. Acrolein is a major cigarette-related lung cancer agent: Preferential binding at p53 mutational hotspots and inhibition of DNA repair. Proc Natl Acad Sci. 2006; 103(42): 15404–15409. 10.1073/pnas.0607031103 17030796 PMC1592536

[10] Geiss O, Bianchi I, Barrero-Moreno J. Correlation of volatile carbonyl yields emitted by e-cigarettes with the temperature of the heating coil and the perceived sensorial quality of the generated vapours. Int J Hyg Environ Health. 2016; 219(3): 268–277. 10.1016/j.ijheh.2016.01.004 26847410

[11] Zhao T, Shu S, Guo Q, Zhu Y. Effects of design parameters and puff topography on heating coil temperature and mainstream aerosols in electronic cigarettes. Atmos Environ. 2016; 134:61–69.

[12] Chen W, Wang P, Ito K, Fowles J, Shusterman D, Jaques PA, et al Measurement of heating coil temperature for e-cigarettes with a "top-coil" clearomizer. PLoS One. 2018; 13(4): e0195925 10.1371/journal.pone.0195925 29672571 PMC5908153

[13] Kasap SO. Principles of Electronic Materials and Devices. 3rd ed Mc-Graw Hill 2006.

[14] Robinson RJ, Hensel EC, Morabito PN, Roundtree KA. Electronic cigarette topography in the natural environment. PLoS One. 2015; 10(6): e0129296 10.1371/journal.pone.0129296 26053075 PMC4460076

[15] Garner C, Stevens RD, Tayyarah R. 2014 Electronic Cigarette Aerosol Parameters Study. Technical Report. E-Cigarette Task Force. 2015

[16] Behar RZ, Hua M, Talbot P. Puffing topography and nicotine intake of electronic cigarette users. PloS One. 2015; 10(2): e0117222 10.1371/journal.pone.0117222 25664463 PMC4321841

[17] Norton KJ, June KM, O’Connor RJ. Initial puffing behaviors and subjective responses differ between an electronic nicotine delivery system and traditional cigarettes, Tob Induc Dis. 2014; 12(1): 17 10.1186/1617-9625-12-17 25324711 PMC4199458

[18] Baassiri M, Talih S, Salman R, Karaoghlanian N, Saleh R, Hage REI, et al Clouds and “throat hit”: Effects of liquid composition on nicotine emissions and physical characteristics of electronic cigarette aerosols. Aerosol Sci Technol of Electronic Cigarettes. 2017; 51(11): 1231–1239. 10.1080/02786826.2017.1341040 PMC7453347 32863527

[19] https://veppocig.com/pages/pg-vs-vg-eliquid. July 07, 2018.

[20] http://www.licensetovape.com/e-liquid-ingredients. July 07, 2018.

[21] https://faculty.missouri.edu/~glaserr/2050f05/Boiling_Diagrams.pdf.

[22] Lendvay AT, Laszlo TS. Cigarette peak coal temperature measurements, The Journal of BTFI GmBH. 1974; 7(5): 276–281.

[23] Sleiman M, Logue JM, Montesinos VN, Russell ML, Litter MI, Gundel LA, et al Emissions from electronic cigarettes: key parameters affecting the release of harmful chemicals. Environ Sci Technol. 2016; 50(17): 9644–9651. 10.1021/acs.est.6b01741 27461870

