# Supplementary figures and images for: Accuracy of commercial electronic nicotine delivery systems (ENDS) temperature control technology
Source: PLoS One. 2018 Nov 5;13(11):e0206937. doi: 10.1371/journal.pone.0206937 (PMC6218080; doi:10.1371/journal.pone.0206937)

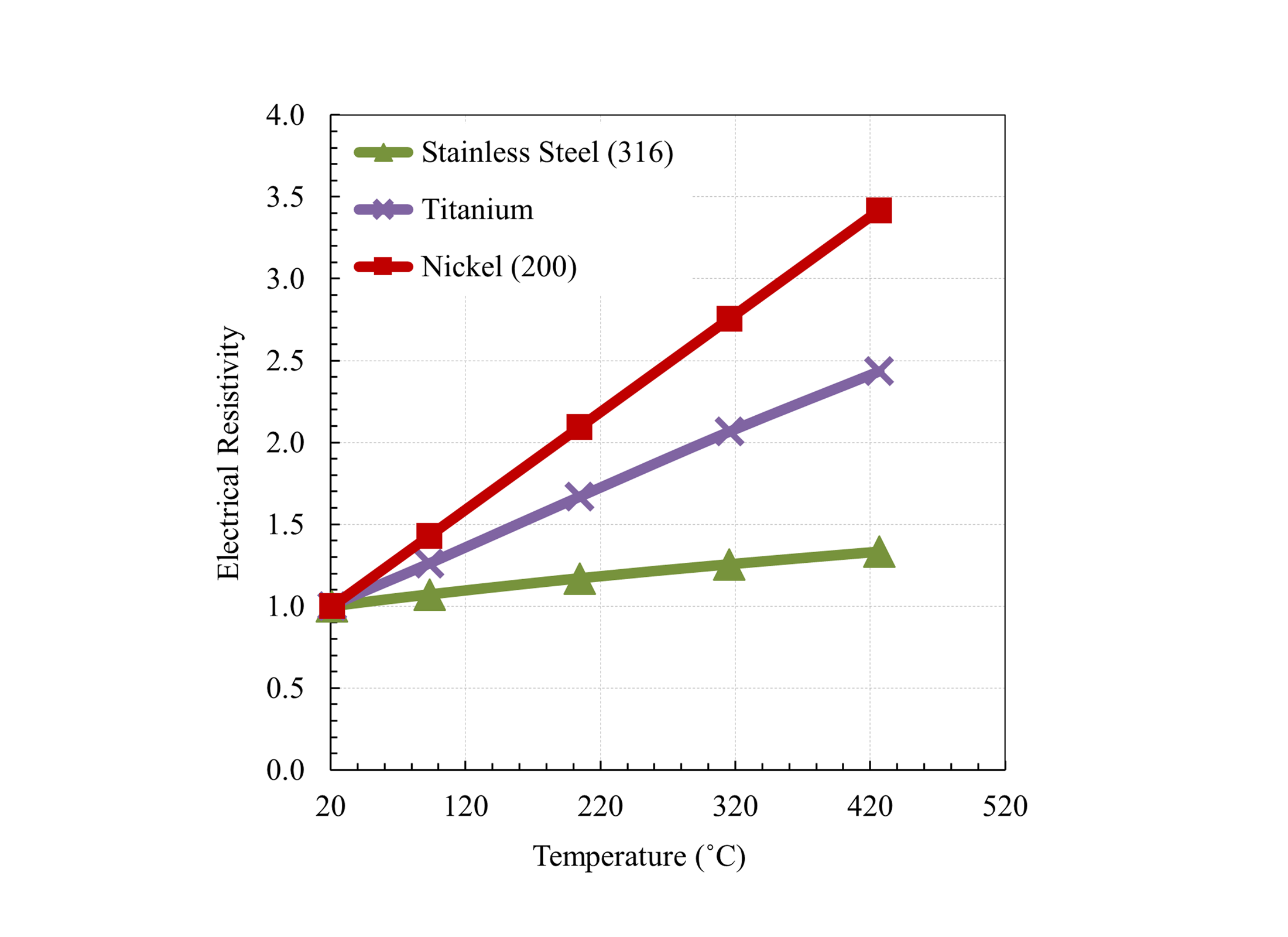

Supplement: S1 Fig — Note: the presented data were collected from the manufacturer website (www.steam-engine.org). (TIF) [file pone.0206937.s001.tif]

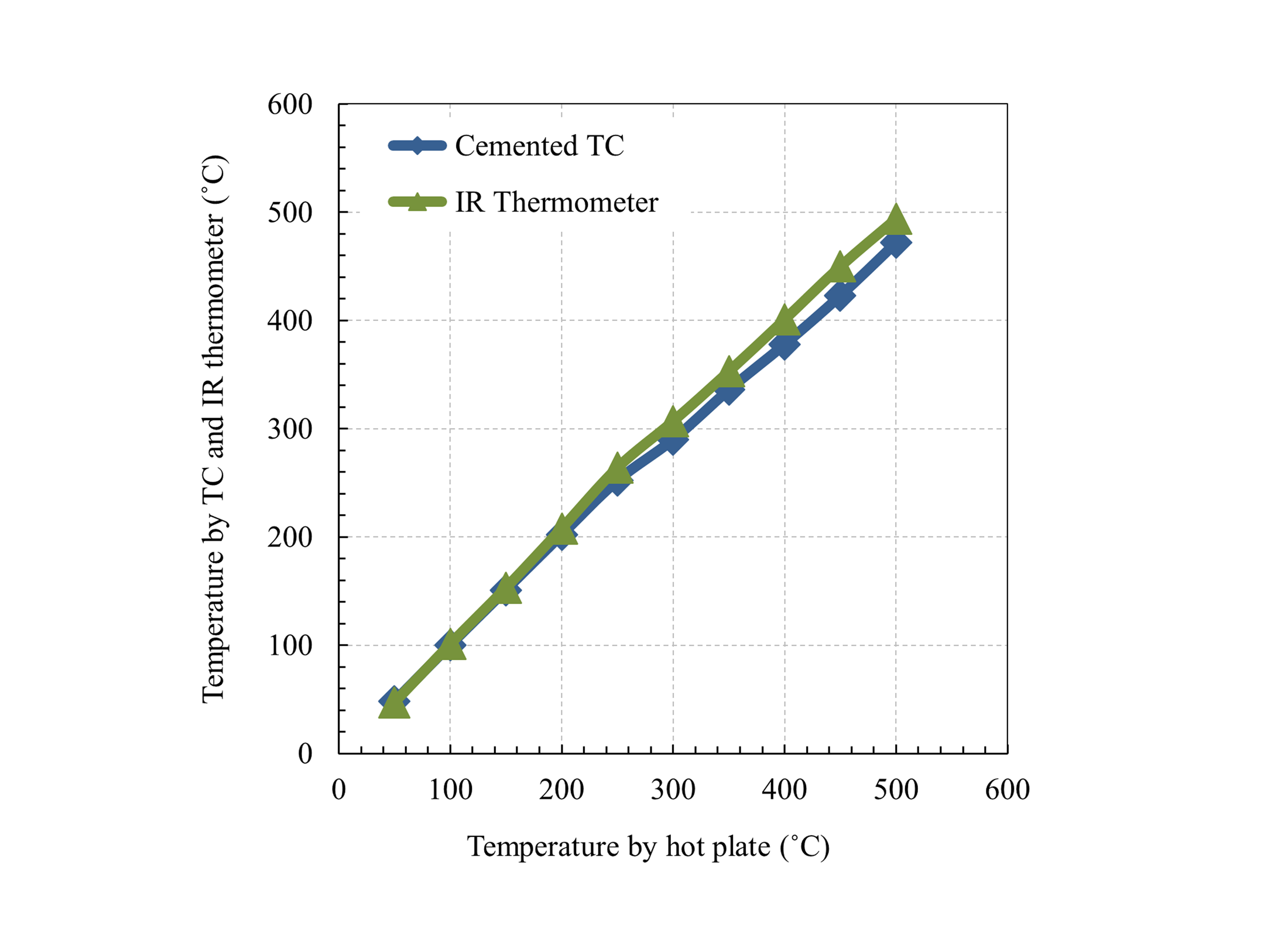

Supplement: S2 Fig — (TIF) [file pone.0206937.s002.tif]

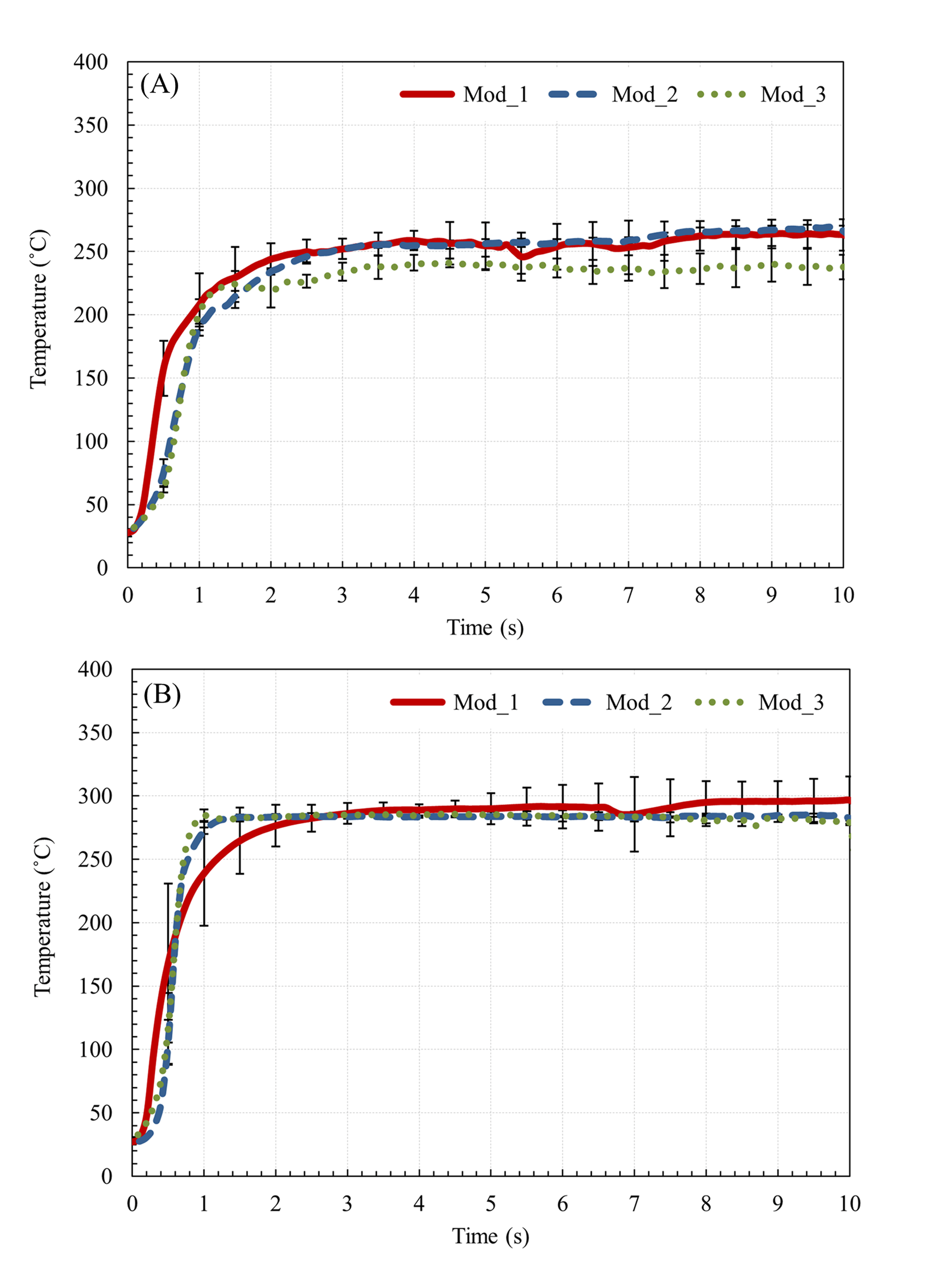

Supplement: S3 Fig — Solid red line: recorded temperature for mod_1, dash blue line: recorded temperature for mod_2, and dot green line: recorded temperature for mod_3. Note: All the three different tested mods (referred to here as mod_1, mod_2, and mod_3) had the same DNA 200 microchip. The power and temperature control were set at 100 W, and 300 °C, respectively. The temperature measurements were made with no airflow for the puff duration of 10 s. (TIF) [file pone.0206937.s003.tif]
