# Supplementary material for: Accuracy of commercial electronic nicotine delivery systems (ENDS) temperature control technology
Source: PLoS One. 2018 Nov 5;13(11):e0206937. doi: 10.1371/journal.pone.0206937 (PMC6218080; doi:10.1371/journal.pone.0206937)
Supplement: S1 Table — Note: An analysis of variance for 5 different time points showed that in 8 of the 10 comparisons, no significant difference was discernable. Essentially, the variation within tests of a single mod exceeded the variation between mods. (DOCX) [file pone.0206937.s004.docx]

| Time | Recorded temperature at the top section (°C) | | | | | | | | | P-value |
| --- | --- | --- | --- | --- | --- | --- | --- | --- | --- | --- |
|  | Mod_1 | | | Mod_2 | | | Mod_3 | | |  |
|  | Trial 1 | Trial 2 | Trial 3 | Trial 1 | Trial 2 | Trial 3 | Trial 1 | Trial 2 | Trial 3 |  |
| 1 s | 179.9 | 225.4 | 219.0 | 193.2 | 188.8 | 188.8 | 189.1 | 206.7 | 208.6 | 0.42 |
| 3 s | 248.9 | 246.1 | 261.3 | 250.5 | 252.8 | 252.2 | 226.3 | 240.4 | 235.2 | 0.02 |
| 5 s | 264.4 | 233.3 | 266.0 | 256.3 | 259.8 | 252.8 | 239.8 | 246.5 | 236.0 | 0.26 |
| 7 s | 251.2 | 233.3 | 275.6 | 261.8 | 257.1 | 258.0 | 248.0 | 234.2 | 228.8 | 0.20 |
| 9 s | 260.3 | 254.7 | 276.6 | 264.1 | 266.5 | 270.7 | 256.7 | 232.1 | 232.1 | 0.04 |
| Time | Recorded temperature in the mid-section (°C) | | | | | | | | | P-value |
|  | Mod_1 | | | Mod_2 | | | Mod_3 | | |  |
|  | Trial 1 | Trial 2 | Trial 3 | Trial 1 | Trial 2 | Trial 3 | Trial 1 | Trial 2 | Trial 3 |  |
| 1 s | 201.8 | 231.8 | 283.2 | 269.7 | 274.7 | 272.8 | 288.6 | 285.7 | 278.9 | 0.13 |
| 3 s | 276.6 | 291.6 | 290.2 | 283.1 | 283.8 | 283.7 | 287.4 | 284.5 | 282.3 | 0.82 |
| 5 s | 292.9 | 276.3 | 300.5 | 283.6 | 283.6 | 284.2 | 288.0 | 283.1 | 284.1 | 0.59 |
| 7 s | 300.3 | 251.6 | 304.7 | 283.0 | 283.1 | 283.8 | 287.9 | 281.0 | 281.7 | 0.98 |
| 9 s | 305.1 | 277.1 | 304.9 | 283.8 | 285.4 | 284.8 | 280.9 | 280.3 | 284.9 | 0.25 |
